# Domestic Service in Institutions

**Published:** 1920-09-18

**Authors:** Gladys M. E. Leigh

**Affiliations:** Nerve Hospital, Edgbaston.


### THE EDITOR'S LETTER-BOX.

### Domestic Service in Institutions.

*To the Editor of* The Hospital.

Sir,—"Cantab's" solution of the domestic problems now so acute in institutional life is unfortunately of little practical value. Every institution now suffers from the daily worker. Usually they decline to commence work before 9 a.m., and there are days when they fail to appear at all; moreover, their appearance is such that in self-defence the institution provides them with caps and overalls laundried on the premises.

The usual wage for a daily worker for 5½ days per week, a working-day consisting of eight hours, is 27s. 6d. weekly; they also receive their midday meal. The suggestion of lady-helps is impractical, as it renders even more acute, if that be possible, the housing problem. Matrons are experiencing sufficient difficulty in providing accommodation for their increasing nursing staffs. Why add to their burden? Also it has been clearly demonstrated lately that women will not take up work which has no economic future.

I can also assure "Cantab" that daily workers take all public holidays with or without permission.—Yours faithfully,

Gladys M. E. Leigh.

Nerve Hospital, Edgbaston.

